# Prevalence of anemia and its associated factors among adult asthmatic patients in Northwest Ethiopia

**DOI:** 10.1186/s12890-023-02501-9

**Published:** 2023-06-21

**Authors:** Yenealem Solomon, Natnael Atnafu Gebeyehu, Getachew Asmare Adella, Gizachew Ambaw Kassie, Misganaw Asmamaw Mengstie, Mohammed Abdu Seid, Endeshaw Chekol Abebe, Molalegn Mesele Gesese, Kirubel Dagnaw Tegegne, Denekew Tenaw Anley, Melkamu Aderajew Zemene, Natnael Moges, Berihun Bantie, Sefineh Fenta Feleke, Tadesse Asmamaw Dejenie, Anteneh Mengist Dessie

**Affiliations:** 1 Department of Medical Laboratory Science, College of Health Sciences, Debre Tabor University, Debre Tabor, Ethiopia; 2 Department of Midwifery, College of Medicine and Health Science, Wolaita Sodo University, Wolaita, Ethiopia; 3 Department of Reproductive Health and Nutrition, School of Public Health, Woliata Sodo University, Sodo, Ethiopia; 4 Department of Epidemiology and Biostatistics, School of Public Health, Woliata Sodo University, Sodo, Ethiopia; 5 Department of Biochemistry, College of Health Sciences, Debre Tabor University, Debre Tabor, Ethiopia; 6 Unit of Physiology, Department of Biomedical Science, College of Health Science, Debre Tabor University, Debre Tabor, Ethiopia; 7 Department of Nursing, College of Medicine and Health Science, Wollo University, Dessie, Ethiopia; 8 Department of Public Health, College of Health Sciences, Debre Tabor University, Debre Tabor, Ethiopia; 9 Department of Pediatrics and Child Health Nursing, College of Health sciences, Debre Tabor University, Debre Tabor, Ethiopia; 10 Department of Comprehensive Nursing, College of Health Sciences, Debre Tabor University, Debre Tabor, Ethiopia; 11 Department of Public Health, College of Health Sciences, Woldia University, Woldia, Ethiopia; 12 Department of Medical Biochemistry, College of Medicine and Health Sciences, University of Gondar, Gondar, Ethiopia

**Keywords:** Asthma, Anemia, Acute exacerbation, Hemoglobin level

## Abstract

**Background:**

Asthma is a heterogeneous disease characterized by chronic airway inflammation. The pathophysiologic processes of asthma can disrupt iron homeostasis, resulting in anemia. However, the association between asthma and anemia among adult asthma patients remains limited. Therefore, the main aim of this study was to determine the prevalence and factors associated with anemia among adult asthmatic patients from May to August 2021.

**Methods:**

An institution-based, cross-sectional study was conducted among 291 asthmatic patients in Northwest Ethiopia. A pre-tested structured questionnaire and checklist were used to collect sociodemographic and clinical data. A blood specimen was collected from asthmatic patients for a complete blood count analysis and morphology assessment. The data were entered into the Epi data software and exported to the statistical package for social science version 20 software for analysis. Non-parametric Mann-Whitney U test was used to compare red blood cell parameters among groups with acute and chronic exacerbations. Binary logistic regression models were used to determine the factors associated with anemia. A p-value less than 0.05 was considered statistically significant.

**Result:**

The overall prevalence of anemia in this study was 11% (95% CI: 7.2–14.8%). Acutely exacerbated asthmatic patients had significantly lower median values of red blood cell parameters such as red blood cell count, hemoglobin, and mean cell hemoglobin when compared to chronic exacerbations. In addition, using systemic corticosteroids (AOR = 4.07, 95% CI: 1.126–14.71, p = 0.032) and being hospitalized in the emergency department (AOR = 3.74, 95% CI: 1.26–11.07, p = 0.017) were found to be significantly associated with anemia.

**Conclusion:**

This study demonstrated that anemia was predominant in adult asthma patients. Red blood cell number, hemoglobin level, and mean corpuscular hemoglobin were significantly lower in acute asthma exacerbations. Therefore, appropriate intervention strategies should be undertaken to reduce the prevalence of anemia among adult asthma patients to reduce further complications and provide better monitoring of asthma patients.

## Introduction

Asthma is a heterogeneous disease characterized by chronic airway inflammation. It is an inflammatory condition associated with airway hyperresponsiveness. Coughing, wheezing, shortness of breath, and chest tightness are common symptoms, along with variable expiratory airflow limitation. These symptoms are intermittent and frequently worse at night or after exercise [1].

Asthma is a significant public health issue [2]. It affects both children and adults. Currently, asthma affects over 339 million people worldwide [3]. The prevalence of asthma is high in developed countries (accounting for 15–20%) [4, 5]. Increasing urbanization, obesity, and environmental pollution are major causes. Although increasing urbanization, smoking, and population growth are factors for the rise in asthma frequency in developing countries [6].

Anemia occurs when the number of red blood cells (RBC), hemoglobin (Hb), or hematocrit value is lower than the normal range [7]. According to the World Health Organization (WHO), anemia is defined as a condition in which the RBC count is insufficient to carry oxygen to meet the body’s physiological needs [8]. A hemoglobin level of less than 13 g/dl for men and 12 g/dl for women is a cut-off value to define anemia regardless of ethnicity [9].

Asthma is known to affect iron homeostasis, resulting in anemia. The pathophysiologic mechanisms of asthma, such as inflammation, bronchoconstriction, and bronchial obstruction disrupt iron homeostasis [10]. Chronic inflammation causes anemia or iron deficiency by impairing the regulation of hepcidin and cytokines, both of which are essential for iron homeostasis [11]. In addition, the involvement of iron in inflammation changes the availability of this metal (Fe^2+^) and activates various pathways that coordinate inflammation [12].

Evidence suggests that anemia is common in asthmatic patients, mainly due to iron deficiency [13]. Lack of iron adversely affects the immune system and pathogen development. It also serves as an independent risk factor for developing lower respiratory tract infections [14]. Therefore, low body iron increases asthmatic attacks. A study by Bucca et al. showed that a decrease in body iron reduces forced expiratory volume (FEV1) [13]. Moreover, iron supplementation might have a role in asthma symptom improvement [15].

On the other hand, hemoglobin can stabilize the oxygen pressure in the blood and tissues. Hence, a lack of iron lowers Hb synthesis. As a result, anemia and reduced Hb levels may be associated with the global rise in asthma and allergic disorders. Anemia is considered a risk factor for asthma exacerbations [16, 17]. Furthermore, anemia decreases the peak expiratory flow (PEF) rate during acute asthma exacerbations [15].

Even though studies reported that hypoxia induces erythropoiesis, different studies have shown that RBC parameters like hemoglobin level, hematocrit, and RBC indices were lower among asthmatic patients. This might be due to the effect of corticosteroids. Use of interferes erythrocyte maturation and erythropoiesis. That predisposes the development of anemia among adult asthma patients [18, 19].

Only a few studies have found highest prevalence of anemia among asthma patients. However, the magnitude of anemia among adult asthmatic patients remains limited. Also, the magnitude of anemia among adult asthma patients in our country including our study area is not studied well. Therefore, our study was an effort to fill this gap and provide information on the burden of anemia among adult asthmatic patients. Furthermore, this study also gives knowledge on variations of RBC parameters with respect to chronic and acute asthma exacerbation states.

## Methods and materials

### Study design, period, and area

The institution-based cross-sectional study was conducted from May to August 2021 in Northwest Ethiopia at Tibebe-Ghion Comprehensive Specialized Hospital (TGCSH) and the University of Gondar Comprehensive Specialized Hospital (UGCSH). TGSH is a teaching hospital located in Northwest Ethiopia, 560 km away from the capital city (Addis Ababa), in the Amhara regional state, Bahirdar city. This hospital provides inpatient and outpatient care for up to 2,000 patients per day. It also serves more than five million people in the catchment area. On the other hand, UGCSH is found in Northwest Ethiopia, in the Amhara Regional State, Gondar town. The hospital serves more than 7 million people in the catchment area. There are over 400 regular follow-ups of asthmatic patients in the outpatient department of this hospital.

### Study population

This study comprised a total of 291 asthmatic patients confirmed by a specialist (working in pulmonary and critical care) via clinical history and lung function tests. Patients had either an acute exacerbation or chronic, stable asthma. On the other hand, asthma patients diagnosed with chronic conditions such as HIV/AIDS, chronic kidney disease, hematological malignancies, blood loss, bleeding disorders, pregnancy, bronchiectasis, and tuberculosis were excluded from this study.

### Sample size determination and sampling technique

#### Sample size determination

The required sample size was determined by using the single population proportion formula. By considering the proportion of 50%, 5% margin error, and 95% confidence interval.

$$n = \frac{{{{\left( {{Z_{\frac{\alpha }{2}}}} \right)}^2}p{\rm{q}}}}{{{{\rm{d}}^2}}} = {\left( {1.96} \right)^2}\left( {0.5 \times 0.5} \right)/{\left( {0.05} \right)^2} = 384$$

**Fig. 1:**
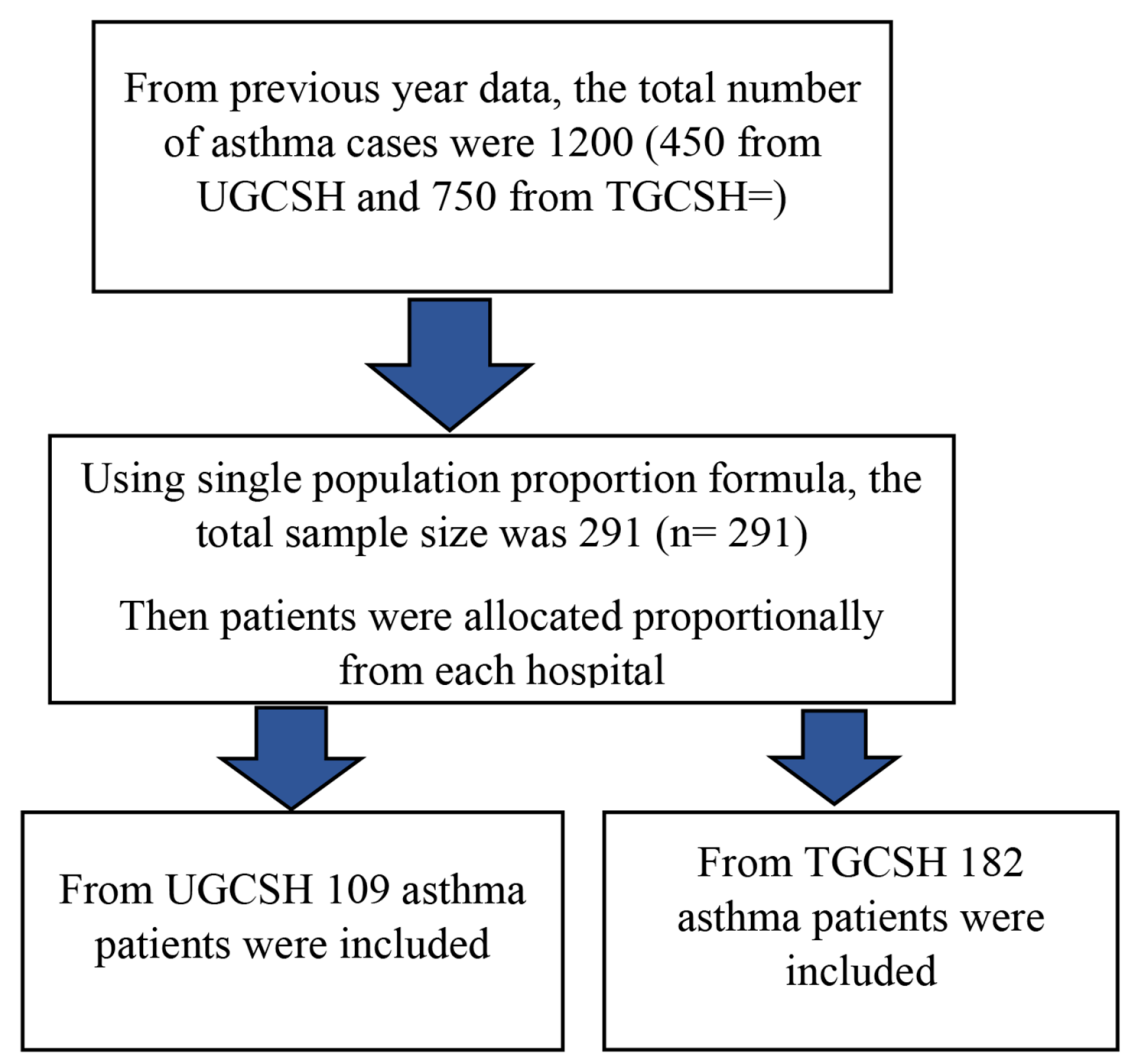
Shows sampling procedure

From the previous annual report, the total number of asthmatic patients in two hospitals per year was 1,200 (N < 10,000). Thus, a reduction formula was used. As a result, the final sample size was 291 (n = 291). The study participants were then proportionally allocated between two hospitals (Fig. 1).

### Sampling technique and procedure

A lottery-based, simple random sampling technique was used to select study participants.

### Operational definitions

- **Asthma**: Any person diagnosed with asthma by a specialist working in the pulmonary and critical care department and who experienced an asthma attack or was treated for asthma in the past 12 months.
- **Asthma-related emergency department admission**: asthmatic patients who were admitted to the emergency room for health care utilization [20].
- **The severity of asthma** was classified based on the Global Initiative for Asthma (GINA) 2019 guideline [21].
- **Stages of asthma** were classified as intermittent, mild persistent, moderate persistent, and severe persistent based on GINA 2019 guidelines [21].
- **A habit of doing physical exercise**: study subjects who had the habit of doing physical activity for at least 20–30 min per day as a continuous activity [22].
- **Acute exacerbation**: asthma patients with decreased expiratory airflow, which can range from mild to a life-threatening state.

### Data collection and sample processing

A structured, pre-tested questionnaire was used to collect socio-demographic and behavioral data from study participants. A checklist was used to collect clinical data from patients’ medical records, such as duration of patients living with asthma, family history of asthma, history of asthmatic drug intake before 3 months, types of asthmatic drugs, chronic disease, severity and stages of asthma, symptoms of asthma, and history of taking other drugs before 3 months. Clinical nurses collected the data through face-to-face interviews and chart reviews (medical record reviews).

A venous blood specimen (4 mL) was collected from asthmatic patients by the trained laboratory technician using the syringe method. Complete blood counts (CBC) of the study participants were analyzed using a five-differential Unicel DxH 800 Coulter cellular analysis system (Beckman Coulter, Ireland). This automated analyzer uses Coulter counting, spectrophotometry, and VCS technology principles.

Peripheral blood morphology examination was performed to confirm flags generated by the automated hematology analyzer, to check the performance of the automated hematology analyzer, and to examine RBC morphology (to classify anemia). A thin blood smear was prepared from leftover blood from the CBC. The smear was air-dried and stained with Wright stain. Then the stained smear was carefully examined with a 100X oil immersion objective by laboratory technologists. All laboratory tests were performed in the UGCSH and TGSH laboratories. The results of each study participant were carefully recorded in a form designed for laboratory result registration.

### Data quality assurance and management

The English-version questionnaire was prepared and then translated into the local language (Amharic). To check for consistency and correctness, the questionnaire was retranslated back into the English version. A pretest was done on 5% of asthmatic patients at the Felege Hiwot Comprehensive Specialized Hospital (FHCSH). FHCSH is located in Northwest Ethiopia in Amhara regional state, Bahirdar city. Based on the findings of the pretest, the questionnaire was modified. In addition, data collectors were trained before starting data collection to minimize technical and observer bias.

Pre-analytical, analytical, and post-analytical phases of quality assurance were maintained in the overall laboratory procedures. Quality control for working equipment and reagents was ensured by using standard controls. Standard operating procedures and manufacturer’s instructions were strictly followed during sample collection and laboratory procedures. The questionnaire was checked for clarity, consistency, and completeness by the principal investigator. The result of every test was properly recorded, reviewed, and transcribed.

### Data processing and statistical analysis

Data was entered into the Epi-data software (version 3.0.4). The data were then cleaned and exported into the Statistical Package for Social Science version 20 software (IBM Corp., Armonk, NY, USA) for analysis. Descriptive statistics, such as frequencies and percentages, were used to summarize the data. The Shapiro-Wilk test and histogram were used to assess normal distribution for continuous variables. Tables and figures were used to present the data.

Since the continuous variables were not normally distributed the non-parametric Mann Whitney U test was used to compare RBC parameters among groups. A bivariable and multivariable binary logistic regression analysis was carried out to determine the association between predictors and outcome variables. The strength of the association was determined by using the crude odds ratio (COR) and adjusted odds ratio (AOR) with a 95% confidence interval (CI). Variables with a p-value of less than 0.2 were fitted into the multivariable logistic regression analysis. Hosmer and Lemeshow’s goodness-of-fit statistic was used to check the model’s fitness. In all cases, a p-value of less than 0.05 was considered statistically significant.

### Ethical consideration

Ethical clearance was gained from the ethical review committee of the School of Biomedical and Laboratory Science, College of Medicine and Health Sciences, University of Gondar, with letter reference number SBMLS-2748/2021. The department of Hematology and Immunohematology provided a support letter, which was forwarded to the directors of UGCSH and TGCSH. In addition, a permission letter to conduct research was obtained from the UGCSH and TGCSH directors.

Written informed consent was obtained from each study participant after the objective of the study was explained. Study participants were identified by using codes rather than individual identifiers. The results and information of the study participants were kept confidential. Study participants who had anemia were linked to the UGCSH and TGSH chronic outpatient departments for proper management. All methods were performed in accordance with the relevant guidelines and regulations (Declaration of Helsinki).

## Result

### Sociodemographic characteristics of the study participants

A total of 291 adult asthma patients were enrolled in this study. The age range was from 18 to 65 years, with a median and interquartile range (IQR) of 50 (38–60) years. Females comprise 164 (56.4%). About 186 (63.9%) of the study participants were urban residents. Regarding educational status, 126 (43.3%) were unable to read and write (Table 1).

**Table 1:** Socio-demographic characteristics of adult asthmatic patients (n = 291)

Variables	Category	Frequency	Percentage %
Age	≤ 40	103	35.4%
	> 40	188	64.6%
Sex	Male	127	43.6%
	Female	164	56.4%
Residence	Urban	186	63.9%
	Rural	105	36.1%
Marital status	Single	38	13.1%
	Married	192	66.0%
	Divorced	27	9.3%
	Widowed	34	11.7%
Education level	Unable to read and write	126	43.3%
	Primary school	64	22.0%
	Secondary school	49	16.8%
	College and above	52	17.9%
Occupation	Housewife	101	34.7%
	Farmer	55	18.9%
	Employer	56	19.2%
	Private worker	57	19.6%
	Non-job including students	22	7.6%
Family size	≤ 4	139	47.8%
	5–8	132	45.4%
	> 8	20	6.9%
Monthly income (ETB)	< 1000	45	15.5%
	1000–5000	191	65.6%
	> 5000	55	18.9%

ETB: Ethiopian Birr

### Behavioral characteristics and body mass index (BMI) of the study participants

Of the study participants, 5 (1.7%) were current cigarette smokers, and 52 (17.9%) had a habit of doing physical exercise. About 64 (22%) had an alcohol-drinking habit. In terms of BMI, 51 (17.5%) were overweight (Table 2).

**Table 2:** Shows behavioral characteristics and BMI of the study participants

Variables	Category	Frequency	Percentage %
Cigarette smoking	Non-smoker	284	97.6%
	Current smoker	5	1.7%
	Ex-smoker	2	0.7%
Alcohol drinking habit	Yes	64	22%
	No	227	78%
Type of alcohol drinker	No drinker	227	78%
	Social drinker	17	5.8%
	Frequent drinker	47	16.2%
Physical exercise	Yes	52	17.9%
	No	239	82.1%
If yes, how often do you exercise?	1–2 times/week	12	23.1%
	3–5 times/week	30	57.7%
	> 5 times/week	10	19.2%
BMI	Normal weight	215	73.9%
	Underweight	25	8.6%
	Overweight	51	17.5%

BMI: body Mass Index

### Clinical characteristics of study participants

The majority of study participants have been living with asthma for less than five years, 121 (41.6%). Around 176 (60.5%) and 186 (63.9%) had moderate severity and moderately persistent stages of asthma, respectively. About 241 (82.8%) were used asthma medication in the past three months. Asthma-related emergency admissions account for 86 (29.6%) (Table 3).

**Table 3:** The clinical characteristics of adult asthmatic patients (n = 291)

Variables	Category	Frequency	Percentage %
Family history of asthma	Yes	88	30.2%
	No	203	69.8%
Duration of patient live with asthma	≤ 5	121	41.6%
	6–10	79	27.1%
	11–15	32	11.0%
	> 15	59	20.3%
Severity of asthma	Mild	34	11.7%
	Moderate	183	62.9%
	Severe	74	25.4%
Stage of asthma	Intermittent	36	12.4%
	Mild persistent	19	6.5%
	Moderate persistent	186	63.9%
	Severe persistent	50	17.2%
Asthma medication	Yes	241	82.8%
	No	50	17.2%
History of taking drugs other than asthma	Yes	91	31.3%
	No	200	68.7%
Comorbidities	Yes	87	29.9%
	No	204	70.1%
**Type of comorbidity**	Hypertension	59	67.8%
	Diabetes mellitus	13	14.9%
	Other#	15	17.2%
ED admission	Yes	86	29.6%
	No	205	70.4%
Asthma drugs	ICS	117	48.5%
	SC	17	7.1%
	SABA	91	37.8%
	Others	16	6.6%
Symptoms of asthma	Shortness of breath	118	40.5%
	Cough	88	30.2%
	Wheezing	13	4.5%

**Note: ICS**: Inhaled Corticosteroids, **SC**: Systemic corticosteroids, **SABA**: Short-acting beta 2 antagonists, **ICS-SABA**: Inhaled Corticosteroids-Short acting beta 2 antagonists **ED**: Emergency department, other# include (epilepsy, polycythemia vera, and bacterial infection)

### Prevalence and severity of anemia among asthmatic patients

The median (IQR) values of RBC, Hb, HCT, and MCV were 4.9 (4.44–5.41), 14.7 (13.4–14.7), 43.4 (40–43.4), and 88.8 (85.1–92.5), respectively (Table 4). In this study, the prevalence of anemia was 11%, with a 95% confidence interval of 7.2–14.8%. In terms of severity, the majority of study participants had mild anemia (82.4%) and about 18% of asthma patients had moderate anemia.

**Table 4:** The median (IQR) of hematological parameters (n = 291)

Parameter	Median (IQR)	Range
RBC	4.9(4.44–5.41)	2.94–7.43
Hb	14.7(13.4–14.7)	8.2–22.9
HCT	43.4(40–43.4)	24.6–68.6
MCV	88.8(85.1–92.5)	76.5–121
MCH	29.7(28.5–31.1)	24.5–37.7
MCHC	33.3(32.6–34.4)	29.1–40.3

**RBC**: Red blood cell; **Hb**: Hemoglobin; **HCT**: Hematocrit; **MCV**: Mean corpuscular volume; **MCH**: Mean cell hemoglobin; **MCHC**: Mean cell hemoglobin concentration; **IQR**: Inter quartile range

### Comparisons of RBC parameters with acute and chronic asthma exacerbations

Since the RBC parameters were not normally distributed, the non-parametric Mann-Whitney U test was used to compare the median (IQR) of RBC parameters between acute and chronic asthma exacerbations. Based on this analysis, RBC, Hb, and MCH showed a statistically significant median difference between acute and chronic asthma exacerbations (Table 5).

**Table 5:** Comparisons of RBC parameters with acute versus chronic asthma exacerbations (Mann-Whitney U test)

Parameter	Acute exacerbations Median (IQR)	Chronic exacerbation Median (IQR)	P- value
RBC	4.87(3.88–5.42)	4.91(4.54–5.4)	0.035*
Hgb	14.6(12.3 − 15.4)	14.8(13.6–16.25)	0.002*
HCT	43.45(33.05–47.12)	43.4(40.45–47.35)	0.10
MCV	87.75(83.6–92.35)	89.3(85.5–92.6)	0.055
MCH	29.4(27.87–30.6)	29.9(28.8–31.3)	0.017*
MCHC	33.05(32.38–34.4)	33.4(32.7–34.5)	0.13

**RBC**: Red blood cell; **Hb**: Hemoglobin; **HCT**: Hematocrit; **MCV**: Mean corpuscular volume; **MCH**: Mean cell hemoglobin; **MCHC**: Mean cell hemoglobin concentration; **IQR**: Inter quartile range *shows statistically significant association

### Factors associated with anemia among adult asthmatic patients

A bivariable and multivariable logistic regression analysis was done to determine factors associated with anemia. In a bivariable logistic regression analysis, variables like monthly income, asthma severity, asthma-related emergency admissions, and SC use showed an association with anemia. While in multivariable logistic regression analysis, taking SC (AOR = 4.07; 95% CI: 1.126–14.71, p = 0.032) and being admitted to the emergency department (AOR = 3.74; 95% CI: 1.26–11.07, p = 0.017) only showed a statistically significant association (Table 6).

**Table 6:** Bi-variable and multivariable logistic regression analysis for factors associated with anemia

Variables	Category	Anemia n (%)		COR (95%CI)	P-value	AOR (95%CI)	P- value
		Yes	No				
Age	≤ 40	8(7.8%)	95(92.2%)	1	0.197	1	0.168
	> 40	24(12.8%)	164(87.2%)	1.74(0.75– 4.02)		2.29(0.7– 7.46)	
Gender	Male	18(14.2%)	108(85.8%)	1	0.13	1	0.325
	Female	14(8.5%)	150(91.5%)	0.56(0.27– 1.18)		0.62(0.24– 1.6)	
Monthly income (ETB)	< 1000	4(12.5%)	41(91.1%)	0.35(0.104– 1.17)	0.028	0.51(0.096– 2.72)	0.43
	1000–5000	16(50%)	175(91.6%)	0.33(0.14– 0.74)		0.66(0.215– 2.03)	0.47
	> 5000	12(21.8%)	43(78.2%)	1		1	
Severity	Mild	12(20.3%)	47(79.7%)	1	0.032	1	
	Moderate	17(9.7%)	159(90.3%)	0.42(0.19– 0.94)		0.497(0.14– 1.8)	0.288
	Severe	5(6.4%)	51(93.6)	0.22(0.059– 0.83)		0.34(0.06– 1.88)	0.217
SC use	Yes	6(35.3%)	11(64.7%)	6.24(2.07– 18.85)	0.001	4.07(1.126– 14.71)	0.032*
	No	18(8%)	206(92%)	1		1	
ICS use	Yes	7(6%)	110(94%)	2.497 (0.99–6.26)	0.051	0.84(0.26– 2.696)	0.77
	No	17(13.7%)	107(86.3%)	1		1	
ED admission	Yes	20(23.3%)	66(76.7%)	4.87(2.26– 10.51)	0.0001	3.74(1.26– 11.07)	0.017*
	No	12(5.9%)	193(94.1%)	1		1	

**Note**: **ED**: Emergency Department, **ICS**: Inhaled Corticosteroids **SC**: Systemic Corticosteroids **COR**: Crude Odds Ratio, **AOR**: Adjusted Odds Ratio, **ETB**: Ethiopian Birr, *statistically significant association, ^1^Reference group

## Discussion

In this study, the prevalence of anemia was 11% (95% CI = 7.2–14.8%). This finding was in line with a study conducted in Egypt (11.2%) [23] and a study done by John et al. (10%) [24]. In addition, different investigations have also revealed the manifestation of anemia in bronchial asthmatic patients [25]. Similarly, different studies reported that the prevalence of anemia was high in asthmatic patients [26, 27].

On the other hand, the findings of this study were lower than a study conducted in Iraq [15]. The discrepancy might be due to the difference in socioeconomic status, geographical location, and sample size (291 vs. 100). Conversely, a study conducted in Ethiopia reported that asthmatic patients had a higher mean RBC count and Hb level than normal healthy controls. But the mean difference was not statistically significant [28]. The study suggested that pulmonary hypoxia in bronchial asthma contributes to the release of erythropoietin hormone from the kidney [29]. The possible explanation for the discrepancy might be due to the difference in study groups. The study by Hailemaryam et al., does not have included patients on corticosteroid therapy. Corticosteroids have effect on erythrocyte production and maturation [19].

There are different ways to explain the existence of anemia in asthma patients. Chronic inflammation, which is a characteristic feature of asthma, may be associated with anemia. It can cause anemia of inflammation by impairing hepcidin regulation. The involvement of cytokines and inflammatory mediators plays an important role in the development of anemia by altering iron availability and ferritin accumulation. Several cytokines, which are essential for iron homeostasis, accelerate the turnover of red blood cells (RBC) and reduce Hb and iron levels [30–32]. Cytokines such as TNFα, IFNγ, or IL-6 can decrease the synthesis and lifespan of RBCs or enhance the phagocytosis of erythrocytes [33–35]. IL-6 stimulates the production of hepcidin by hepatocytes, which disrupts iron homeostasis [36, 37].

In particular, anemia of inflammation can be related to aging. Studies have shown that older age might be a risk factor for inflammatory anemia [38, 39]. In the present study, the majority of participants were elderly (age > 45 years). Of those older patients, about 24 (75%) developed anemia. Therefore, older asthmatic patients may be more likely to develop anemia than younger patients.

Based on the non-parametric Mann-Whitney U test, patients with acute exacerbations had significantly lower median (IQR) values of RBC, Hb, and MCH when compared to patients with chronic asthma exacerbations. This might be due to the fact that asthma disrupts iron hemostasis. Thus, disruption of iron hemostasis leads to a decrease in the amount of metal (Fe^2+^) necessary for hemoglobin synthesis [10, 12]. Therefore, RBC production is reduced due to impaired hemoglobin synthesis. On the other hand, anemia may exaggerate the inflammatory process in asthma. Indeed, increased inflammation aggravates acute exacerbations [25]. The presence of anemia and low iron levels may be associated with lower PEF, particularly in acute exacerbations. Therefore, patients with anemia might have more severe asthma episodes (acute exacerbations).

Asthmatic patients who were admitted to the emergency department had 3.7 times higher odds of having anemia than patients who had not been admitted to the emergency department (AOR = 3.74; 95% CI; 1.26–11.07; p = 0.017). This might be due to the fact that anemic patients may have a higher risk of developing more asthma attacks, which cause emergency admissions. In asthma patients, the presence of anemia can be associated with more severe clinical patterns (poorly controlled asthma) [15]. Moreover, anemia can exaggerate inflammation in asthma patients [25].

Study participants who were using SC had four times higher odds of developing anemia than those who had not taken SC (AOR = 4.07; 95% CI: 1.126–14.71, p = 0.032). The possible explanation might be due to the effects of drugs. A study by Saidu H et al. reported the decreased HCT, Hb, and RBC values of asthmatic patients on corticosteroids compared with healthy controls [18]. It has been reported that corticosteroids have a direct impact on erythropoietin signaling and transcriptional processes to affect the maturation of erythroid cells [19]. In addition, corticosteroids may affect the metabolism of iron and inhibit erythropoiesis [19]. When given parenterally, corticosteroids have the strongest overall effects [40]. As a result, these general effects of corticosteroids on erythropoiesis might be associated with the occurrence of anemia among adult asthmatic patients.

This study has many limitations. Firstly, the cross-sectional nature of the study does not employ cause-and-effect relationships (temporal relationships). Another limitation is due to the lack of testing materials, we were unable to assess iron parameters like serum iron level and ferritin level (we have not investigated iron deficiency anemia) and inflammatory cytokines like IL-6 and TNFα.

## Conclusion

In conclusion, our study indicated the predominant existence of anemia among adult asthmatic patients. Asthma-related emergency department admissions and SC use were associated with anemia among adult asthma patients. Therefore, appropriate intervention strategies should be implemented to lower the prevalence of anemia among adult asthma patients to reduce further complications, hospitalization, and provide better follow-up of asthma patients. Furthermore, the effect of asthmatic drugs such as ICS and SC on erythropoiesis should be independently assessed with an appropriate control group in the future.

## Data Availability

All the data on which the conclusions of this manuscript were drawn are available from the corresponding author. As a result, anyone who needs the data can obtain it upon reasonable request.

## Abbreviations

AOR: Adjusted Odds Ratio
BMI: Body Mass Index
CI: Confidence Interval
COR: Crude Odds Ratio
ED: Emergency Department
FEV: Forced Expiratory Volume FHCH: Felege Hiwot Specialized Hospital
FHCH: Felege Hiwot Specialized Hospital
GINA: Global Initiative for Asthma
Hb: hemoglobin
HCT: Hematocrit
ICS: Inhaled Corticosteroids
IQR: interquartile range
PEF: peak expiratory flow
RBC: red blood cell
MCH: Mean Cell Hemoglobin
MCHC: Mean Corpuscular Hemoglobin Concentration
MCV: Mean Corpuscular Volume
SC: systemic corticosteroids
SABA: short-acting beta-2 antagonists
TGSH: Tibebe-Gihon Specialized Hospital
UGCSH: University of Gondar Comprehensive Specialized Hospital
WHO: World Health Organization

## References

[1] World Health organization (WHO). Asthma fact sheet. Accessed in November; 2022. https://www.who.int/news-room/fact-sheets/detail/asthma.

[2] Dharmage SC, Perret JL, Custovic A (2019). Epidemiology of asthma in children and adults. Front Pead.

[3] Global Asthma Network (GAN). The Global Asthma Report 2018. Auckland, New Zealand 2018. www.Global asthma network. org. Accessed in November 2022.

[4] Sears MR (2014). Trends in the prevalence of asthma. Chest.

[5] Baïz N, Annesi-Maesano I. Is the asthma epidemic still ascending? Clinics in chest medicine. 2012;33(3):419–29. 10.1016/j.ccm.2012.06.001 22929092

[6] Adeloye D, Chan KY, Rudan I, Campbell H (2013). An estimate of asthma prevalence in Africa: a systematic analysis. Croatian Med J.

[7] WHO. Haemoglobin concentrations for the diagnosis of anaemia and assessment of severity. Vitamin and Mineral Nutrition Information System. Geneva, World Health Organization, 2011 WHO/NMH/NHD/MNM/11.1. http://www.who.int/vmnis/indicators/haemoglobin. Accessed in November, 2022

[8] World Health Organization. Anaemia. Available at: https://www.who.int/health-topics/anaemia#tab=tab_1. Accessed on December 02, 2022.

[9] World Health Organization. Haemoglobin concentrations for the diagnosis of anaemia and assessment of severity: Vitamin and Mineral Nutrition Information System 2011. https://www.who.int/vmnis/indicators/haemoglobin.pdf.

[10] Ghio AJ (2016). Asthma as a disruption in iron homeostasis. Biometals.

[11] Chang J-E, Lee H-M, Kim J, Rhew K (2021). Prevalence of Anemia in Pediatric Patients according to Asthma Control: propensity score analysis. J Asthma Allergy.

[12] Nakagawa H, Tamura T, Mitsuda Y, Goto Y, Kamiya Y, Kondo T (2014). Inverse correlation between serum interleukin-6 and iron levels among japanese adults: a cross-sectional study. BMC Hematol.

[13] Bucca C, Culla B, Brussino L, Ricciardolo F, Cicolin A, Heffler E (2012). Effect of iron supplementation in women with chronic cough and iron deficiency. Int J Clin Pract.

[14] Ramakrishnan K, Harish P (2006). Hemoglobin level as a risk factor for lower respiratory tract infections. Indian J Pediatr.

[15] Al-Tameemi W, Habeeb R (2020). Impact of anemia in patient with asthma. Hematol Transfus Int J.

[16] Guyton AC, Hall JA. Effect of hemoglobin to buffer the tissue PO2. In: Text Book of Medical Physiology 12th ed. Philadelphia.

[17] Ganong WP. Gas transport between the lung and the tissues. In: Review of Medical Physiology, 26th ed. New York.

[18] Saidu H, Iyanda R, Garba N, Danladi S, Aliyu I, Bala J (2021). Hematological profiles of nigerian patients with asthma on inhaled Corticosteroids. Ibom Med J.

[19] Stellacci E, Di Noia A, Di Baldassarre A, Migliaccio G, Battistini A, Migliaccio AR (2009). Interaction between the glucocorticoid and erythropoietin receptors in human erythroid cells. Exp Hematol.

[20] Stridsman C, Axelsson M, Warm K, Backman H (2021). Uncontrolled asthma occurs in all GINA treatment steps and is associated with worse physical health–a report from the OLIN adult asthma cohort. J Asthma.

[21] Global Initiative for Asthma. Global Strategy for Asthma Management and Prevention., 2019. Global Initiative for Asthma (GINA). Available at http://www.ginasthma.org. 2019; Accessed in: October 20, 2022.

[22] Corbridge SJ, Nyenhuis SM (2017). Promoting physical activity and exercise in patients with asthma and chronic obstructive pulmonary disease. J nurse practitioners.

[23] Abdelaziz AO, Abd E-HAE-H, Makram O, Abd El-Aziz MO, Magdy ME-H, El-Sharkawy E (2018). Prevalence and impact of anemia in patients with chronic respiratory diseases. Egypt J Chest Dis Tuberculosis.

[24] John M, Lange A, Hoernig S, Witt C, Anker SD (2006). Prevalence of anemia in chronic obstructive pulmonary disease: comparison to other chronic diseases. Int J Cardiol.

[25] Rashid HM, Khan M, Jamal M, Shah W (2019). Anemia in Asthmatic Females exaggerates the severity of inflammation in Asthma by Inducing Dyslipidemia, High levels of IgE and Absolute Eosinophil count: role of anemia on female asthmatic patient’s lipidemia, IgE and eosinophil count. Proc Pakistan Acad Sciences: Part B (Life Environ Sciences).

[26] Rhew K, Brown JD, Oh JM (2020). Atopic disease and anemia in korean patients: cross-sectional study with propensity score analysis. Int J Environ Res Public Health.

[27] Rhew K, Oh JM (2019). Association between atopic disease and anemia in pediatrics: a cross-sectional study. BMC Pediatr.

[28] Hailemaryam T, Adissu W, Gedefaw L (2018). Hematological profiles among asthmatic patients in southwest ethiopia: a comparative cross? Section study. Hematol Transfus Int J.

[29] Ge R-L, Witkowski S, Zhang Y, Alfrey C, Sivieri M, Karlsen T (2002). Determinants of erythropoietin release in response to short-term hypobaric hypoxia. J Appl Physiol.

[30] Weiss G, Ganz T, Goodnough LT (2019). Anemia of inflammation. Blood. J Am Soc Hematol.

[31] Nemeth E, Valore EV, Territo M, Schiller G, Lichtenstein A, Ganz T (2003). Hepcidin, a putative mediator of anemia of inflammation, is a type II acute-phase protein. Blood The Journal of the American Society of Hematology.

[32] Theurl I, Mattle V, Seifert M, Mariani M, Marth C, Weiss G (2006). Dysregulated monocyte iron homeostasis and erythropoietin formation in patients with anemia of chronic disease. Blood.

[33] Morceau F, Dicato M, Diederich M. Pro-inflammatory cytokine-mediated anemia: regarding molecular mechanisms of erythropoiesis. Mediators of inflammation. 2009;2009. 10.1155/2009/405016 PMC2830572 20204172

[34] Libregts SF, Gutiérrez L, de Bruin AM, Wensveen FM, Papadopoulos P, van Ijcken W (2011). Chronic IFN-γ production in mice induces anemia by reducing erythrocyte life span and inhibiting erythropoiesis through an IRF-1/PU. 1 axis. Blood The Journal of the American Society of Hematology.

[35] Milner JD, Orekov T, Ward JM, Cheng L, Torres-Velez F, Junttila I (2010). Sustained IL-4 exposure leads to a novel pathway for hemophagocytosis, inflammation, and tissue macrophage accumulation. Blood The Journal of the American Society of Hematology.

[36] Nemeth E, Rivera S, Gabayan V, Keller C, Taudorf S, Pedersen BK (2004). IL-6 mediates hypoferremia of inflammation by inducing the synthesis of the iron regulatory hormone hepcidin. J Clin Investig.

[37] Rodriguez R, Jung C-L, Gabayan V, Deng JC, Ganz T, Nemeth E (2014). Hepcidin induction by pathogens and pathogen-derived molecules is strongly dependent on interleukin-6. Infect Immun.

[38] Wawer AA, Jennings A, Fairweather-Tait SJ (2018). Iron status in the elderly: a review of recent evidence. Mech Ageing Dev.

[39] Vanasse GJ, Berliner N (2010). Anemia in elderly patients: an emerging problem for the 21st century. Hematol 2010 Am Soc Hematol Educ Program Book.

[40] Ericson-Neilsen W, Kaye AD (2014). Steroids: pharmacology, complications, and practice delivery issues. Ochsner J.

